# Differential Responses of Plant Primary Productivity to Nutrient Addition in Natural and Restored Alpine Grasslands in the Qinghai Lake Basin

**DOI:** 10.3389/fpls.2021.792123

**Published:** 2021-12-20

**Authors:** Chunli Li, Yonghui Li, Xinwei Li, Li Ma, Yuanming Xiao, Chunhui Zhang

**Affiliations:** ^1^State Key Laboratory of Plateau Ecology and Agriculture, Qinghai University, Xining, China; ^2^Key Laboratory of Cold Regions Restoration Ecology, Northwest Institute of Plateau Biology, Chinese Academy of Science, Xining, China; ^3^Key Laboratory of Tibetan Medicine Research, Northwest Institute of Plateau Biology, Chinese Academy of Science, Xining, China

**Keywords:** alpine grassland, plant primary productivity, nitrogen and phosphorus additions, restored grassland, climate change

## Abstract

Climate, land-use changes, and nitrogen (N) deposition strongly impact plant primary productivity, particularly in alpine grassland ecosystems. In this study, the differential responses of plant community primary productivity to N and phosphorus (P) nutrient application were investigated in the natural (NG) and “Grain for Green” restored (RG) alpine grasslands by a continuous 3-year experiment in the Qinghai Lake Basin. N addition only significantly promoted plant aboveground biomass (AGB) by 42% and had no significant effect on belowground biomass (BGB) and total biomass (TB) in NG. In comparison with NG, N addition elevated AGB and BGB concurrently in RG by 138% and 24%, respectively, which further significantly increased TB by 41% in RG. Meanwhile, N addition significantly decreased BGB and the AGB ratio (R/S) both in NG and RG. Compared with N addition, P addition did not perform an evident effect on plant biomass parameters. Additionally, AGB was merely negatively influenced by growing season temperatures (GST) under the N addition treatment in NG. AGB was negatively associated with GST but positively related to growing season precipitation (GSP) in RG. By contrast, changes in the R/S ratio in RG were positively correlated with GST and negatively related to GSP. In sum, the results revealed that plant community biomass exhibited convergent (AGB and R/S) and divergent (BGB and TB) responses to N addition between NG and RG. In addition, the outcomes suggested that climate warming would enhance plant biomass allocation to belowground under ongoing N deposition, and indicated the significance of precipitation for plant growth and AGB accumulation in this restored alpine grassland ecosystem.

## Introduction

As a representation of primary productivity and carbon (C) uptake by plants, biomass is a foundational measure that can be used to infer aspects of nutrient cycling and energy flow in terrestrial ecosystems (Borer et al., 2014; Fay et al., 2015; Grace et al., 2016; Kohli et al., 2019). Accounting for approximately one-third of the Earth's terrestrial net primary production, the plant community biomass is central to understanding the structure and functioning of grasslands, and is vital for the C storage and biogeochemical cycling of ecosystems (Hoekstra et al., 2005; Luyssaert et al., 2008; Borer et al., 2014; Erb et al., 2018; Zhang and Xi, 2021). Humans have heavily impacted grasslands, and more than two-thirds of their biomass has been converted to human-dominated uses (primarily agriculture; Borer et al., 2014; Fay et al., 2015).

Plant growth and biomass allocation are susceptible to climatic change and are limited by complex and multiple resource availabilities, such as soil nutrients, water, and light (Hoekstra et al., 2005; Elser et al., 2007; LeBauer and Treseder, 2008; Poorter et al., 2012; Borer et al., 2014). Plant community productivity and soil C sequestration of grassland ecosystems are widely accepted to be nutrient limited, largely depending on the bioavailability of nitrogen (N) and phosphorus (P) (Elser et al., 2007; Ågren et al., 2012; Fay et al., 2015; Wieder et al., 2015). The availability of N has been deemed an essential determinant of the aboveground net primary productivity (ANPP) across terrestrial ecosystems, and N fertilization could promote plant N uptake and ultimately improve plant biomass production (Elser et al., 2007; LeBauer and Treseder, 2008; Borer et al., 2014; Fay et al., 2015; Wang et al., 2020). However, nutrient co-limitation of grassland productivity is common and more widely recognized (Ågren et al., 2012; Borer et al., 2014; Fay et al., 2015; Wang et al., 2020).

Land-use transitions, as the most intensive anthropogenic interference, alter plant coverage, composition, and productivity directly (Lal, 2002; Sartori et al., 2007; Jelinski and Kucharik, 2009; Li et al., 2016a,b). Generally, cultivation results in massive losses of C and N from the soil; with plant restoration on cropped land, soil nutrient content can be recovered to some extent (Jelinski and Kucharik, 2009; Li et al., 2016a, 2019). Associated with human activities, such as the heavy use of inorganic fertilizers in the process of farming, human-induced N and P deposition has continued to increase during the past several decades worldwide (Galloway et al., 2008; Phoenix et al., 2012). This phenomenon has caused a substantial increase in N and P bioavailability. These element depositions supply an important potential nutrient source for maintaining grassland primary productivity, which would be beneficial to the growth of plant community (Fay et al., 2015; Stevens et al., 2015; Zhu et al., 2016; Wang et al., 2020); however, the additional nutrient input might have adverse effects on plant community composition, structure, and alter ecosystem functionality (Phoenix et al., 2012; Penuelas et al., 2013; Zhu et al., 2016). In addition, the ongoing but imbalanced element depositions potentially induce limitation by other resources (Penuelas et al., 2013; Grace et al., 2016). Numerous studies have shown that N deposition could promote grassland ANPP, and highlighted the importance of N and P synergistic co-limitation in grasslands (Borer et al., 2014; Fay et al., 2015; Grace et al., 2016; Zhao et al., 2019; Wang et al., 2020). N and P fertilizers have received considerable attention in grassland restoration (Fay et al., 2015; Cerasoli et al., 2018; Wang et al., 2018, 2020). However, to date, previous researches have focused primarily on natural grasslands (Borer et al., 2014; Fay et al., 2015; Grace et al., 2016; Zhao et al., 2019). The consequences and effects of anthropogenic nutrient inputs (N and P) vary according to land use, and this remains poorly understood on restored grasslands. It is crucial for potential C sequestration in terrestrial ecosystems and is challenging to predict.

Owing to its ecological uniqueness in Eurasia, the alpine grassland of the Qinghai-Tibetan Plateau (QTP) is critical to global C sequestration and the development of regional husbandry (Shen et al., 2014; Wei et al., 2019; Dong et al., 2020). Plant growth and productivity are ordinarily limited by soil available N and P, resulting from the low temperatures and high altitude in the QTP (Jiang et al., 2013; Fu et al., 2015; Fu and Shen, 2016a,b). As the main alpine grassland area of Qinghai province, the Qinghai Lake Basin (QLB) is a distinctive geographical unit within the QTP. The ecosystem's internal fragility, coupled with external environmental and land-use changes, makes the alpine grasslands in QLB highly sensitive to human activities and ongoing climate change (Cui and Li, 2015; Tao et al., 2021). Therefore, studying the relationships among the environment, ecology, and agriculture/pasture development embedded within climate change scenarios is essential. Because of agriculture and the “Grain for Green” project, native alpine grasslands in the QLB were extensively cultivated to obtain farmland in the mid-1950s; following severe degradation of soil quality, cropped land was returned to grassland in the early 2000s. Previous studies have validated the significant effects of land-use changes on soil C stock patterns and revealed that N and P additions could improve soil quality (Li et al., 2016a, 2019). To date, however, few studies have explored the interaction effects of land use, element depositions, and changes in meteorological factors on the community productivity of the “Grain for Green” grassland (i.e., restored grassland). It is urgent to obtain detailed information about the multiple impacts of land use and nutrient addition on vegetation community primary productivity and biomass allocation between above- and belowground. The results will permit a better understanding of their profound influences on ecosystem productivity and soil C sequestration under a background of global change and accordingly altered climatic and edaphic conditions.

Here, a consecutive 3-year field experiment *in situ* was manipulated in two types of grassland around the QLB, including the natural alpine grassland (NG) and the “Grain for Green” (restored) alpine grassland (RG). The study aimed (1) to determine the presence and magnitude of N and P addition effects on plant community biomass; (2) to investigate the convergent and divergent responses of plant community biomass to nutrient application in the natural and restored grasslands; (3) to explore the influence of interannual meteorological factors on primary productivity and biomass allocation. The findings will provide more detailed information and explanations for nutrient limitations, resulting from land use and climate change scenarios.

## Materials and Methods

### Study Site

The field experiment was carried out in Gangcha County, located on the northern margin of Qinghai Lake and within the northeast part of the QTP. The mean annual temperature in this region is around −0.6°C, and the mean annual precipitation is ~370.5 mm (<400 mm), which occurs almost exclusively in the plant-growing season (from June to August). The soil type of the study site belongs to Dark Chestnut [QPARPO (Qinghai province agricultural resource planning office), 1995]. The two grasslands, NG and RG (37°21′N, 100°04′E, and 3,313 m a.s.l.), both experienced minimal disturbance from human activity and animal grazing in recent years, following the exclusion of livestock grazing by fencing. The NG was dominated by *Stipa purpurea, Kobresia humilis, Elymus nutans, Leymus secalinus*, and *Melissilus ruthenicus*. The RG was used as cropland until the early 2000s but has undergone no grass harvesting, grazing, fertilization, or irrigation since 2002. The constructive grass species of RG was *Elymus nutans*, and other principal plant species included *Elymus nutans, Leymus secalinus*, and *Potentilla anserine* at the present restoration stage.

### Experiment Design

The manipulative experiments were established with a randomized block design in June 2012. Four treatments were included: (1) CK (control, without N or P addition); (2) N, nitrogen addition; (3) P, phosphorus addition; and (4) N × P, a combination of nitrogen and phosphorus addition. N was added in the form of NH_4_NO_3_ at the rate of 10 g N·m^−2^ year^−1^, and P was added in the form of Ca(H_2_PO_4_)_2_·H_2_O at the rate of 5 g P·m^−2^ year^−1^. The addition rates were applied based on the typical amounts frequently used in the QTP alpine grassland (Jing et al., 2016). Six blocks (replicated plots) were established for each treatment. Each plot had 3 × 3-m quadrat areas, and 24 plots were included in each grassland, with a 1-m buffer belt between plots. Nutrient addition was conducted in late June or early July, coinciding with the vigorous growth period of the vegetation. N and P were added one time per year in 2012, 2013, and 2014.

### Field Sampling

Plant materials were collected in middle-late August annually, coinciding with the period of greatest aboveground biomass. One 50 × 50-cm quadrat was randomly placed within each plot. Briefly, all the standing green shoots were cut at the soil surface and oven-dried at 65°C to constant weights to obtain plant community aboveground biomass (AGB). After the aboveground plant and surface litter were removed, the belowground roots were obtained with stainless steel root auger (8 cm diameter) from three random points in the topsoil (0–10 cm, more than 70% root distributed in this soil layer, Li et al., 2016a) of each quadrat. The three subsamples were then mixed as one sample and sealed in a polyethylene bag. After transport to the laboratory, visible root materials were carefully picked out through a 2.-mm sieve, washed, then oven-dried at 65°C to constant mass. This material was used to estimate plant community belowground biomass (BGB). The total plant community biomass (TB) was equal to the sum of AGB and BGB. The ratio of root and shoot (R/S, BGB: AGB) was used to represent the biomass allocation pattern. In brief, plant community biomass parameters include AGB, BGB, TB, and R/S in this study.

### Statistical Analysis

All statistical analyses were analyzed using SPSS 21.0 software (SPSS, Chicago, USA). General linear models and ANOVAs were applied to test the main and interaction effects of nutrient addition and sampling year on AGB, BGB, TB, R/S; a *t-*test was employed to test significant differences of plant community biomass parameters between the two grasslands. Multiple comparisons proceeded using the least standard difference method. For improving normality, data were log-transformed as necessary. Ordinary regression models were built to estimate the effects of climatic factors on plant community biomass parameters using the most powerful explanatory models. Pearson correlation was used to analyze the relationships between biomass parameters. We obtained the plant-growing season (from June to August) temperatures (GST) and precipitation (GSP) data from the China meteorological data sharing service system. All figures were completed using Excel and CorelDraw software.

## Results

### Effects of Nutrient Addition on Plant Community Biomass

The plant community biomass of the alpine grassland was affected by N addition (Table 1). By contrast, no detectable effect of P addition and the interaction of N and P additions were found for all the plant biomass parameters (*p* > 0.05, Table 1). Therefore, the effects of nutrient addition on plant community biomass only referred to N addition hereafter.

**Table 1:** Effects of nitrogen addition (N), phosphorus addition (P), and sampling year (Y) and their interaction effects on plant community AGB, BGB, TB, and R/S in the natural (NG) and restored (RG) grasslands.

		**N**		**P**		**Y**		**N × P**		**N × Y**		**P × Y**		**N × P × Y**	
		***F***	***p***	***F***	***p***	***F***	***p***	***F***	***p***	***F***	***p***	***F***	***p***	***F***	***p***
AGB	NG	**59.762**	**<0.001**	2.751	0.102	**6.389**	**0.003**	3.322	0.073	0.083	0.920	1.332	0.272	0.411	0.655
	RG	**323.95**	**<0.001**	0.985	0.325	**92.213**	**<0.001**	0.024	0.878	**6.643**	**0.002**	0.142	0.868	0.368	0.693
BGB	NG	0.133	0.716	0.005	0.944	2.464	0.094	0.958	0.332	1.417	0.250	0.760	0.472	0.524	0.595
	RG	**12.949**	**<0.001**	3.427	0.069	0.655	0.523	0.026	0.873	1.227	0.300	0.718	0.492	0.599	0.553
TB	NG	0.367	0.547	0.293	0.590	2.214	0.118	1.387	0.244	1.790	0.176	0.977	0.382	0.369	0.693
	RG	**56.444**	**<0.001**	2.890	0.094	**7.675**	**0.001**	0.002	0.965	0.589	0.558	0.654	0.524	0.546	0.582
R/S	NG	**11.549**	**0.001**	0.095	0.759	0.101	0.904	0.145	0.705	1.344	0.268	0.075	0.928	0.678	0.512
	RG	**60.896**	**<0.001**	3.881	0.053	**27.428**	**<0.001**	0.003	0.956	**4.619**	**0.014**	0.358	0.700	0.368	0.694

*AGB, aboveground biomass; BGB, belowground biomass; TB, total biomass; R/S, the ratio of root and shoot. Significant results (p < 0.05) were bolded*.

**Figure 1:**
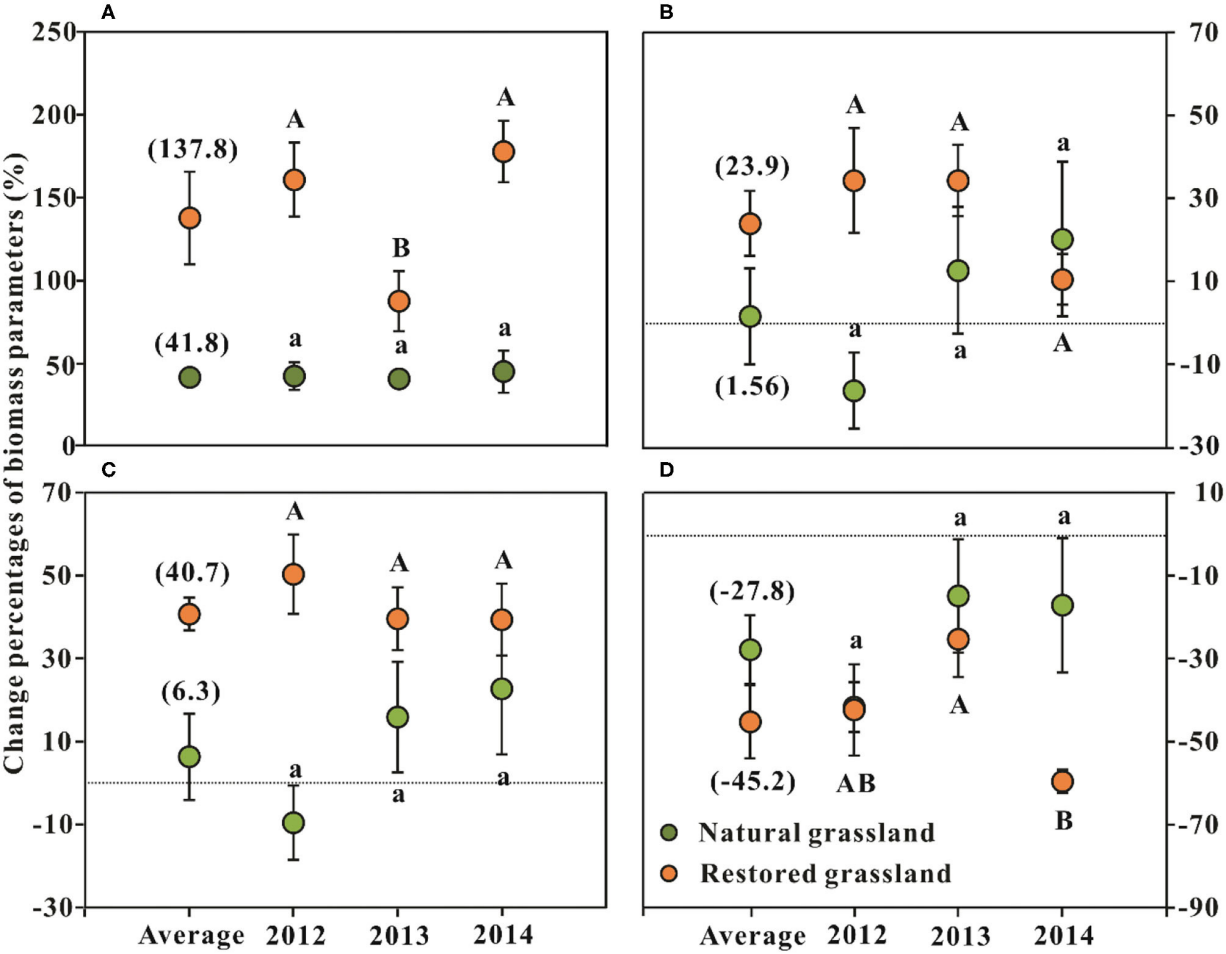
Effects of nitrogen addition on the changes in plant community biomass parameters (mean ± SE, *n* = 6) in the alpine grasslands around Qinghai Lake Basin. **(A)** Aboveground biomass; **(B)** belowground biomass; **(C)** total biomass; **(D)** R/S ratio. R/S = The ratio of root (below-) and shoot (aboveground) biomass; Average represents the average effect across the three sampling years (mean ± SE, *n* = 18). Different lowercase letters indicate significant differences among sampling years in the natural grassland at *p* < 0.05, and uppercase letters indicate significant differences between sampling years in the restored grassland at *p* < 0.05.

Nitrogen N addition significantly increased plant AGB of NG and RG by 42 and 138%, respectively (*p* < 0.001, Table 1; Figure 1A). N addition significantly increased BGB and TB in RG by 24 and 41% (*p* < 0.01, Table 1; Figures 1B,C), respectively; it showed no evident effects on BGB and TB in NG (*p* > 0.05, Table 1; Figures 1B,C). N addition significantly decreased the R/S ratio of NG and RG by 28 and 45%, respectively (*p* < 0.001, Table 1; Figure 1D).

### Comparisons of Plant Community Biomass Between NG and RG

**Figure 2:**
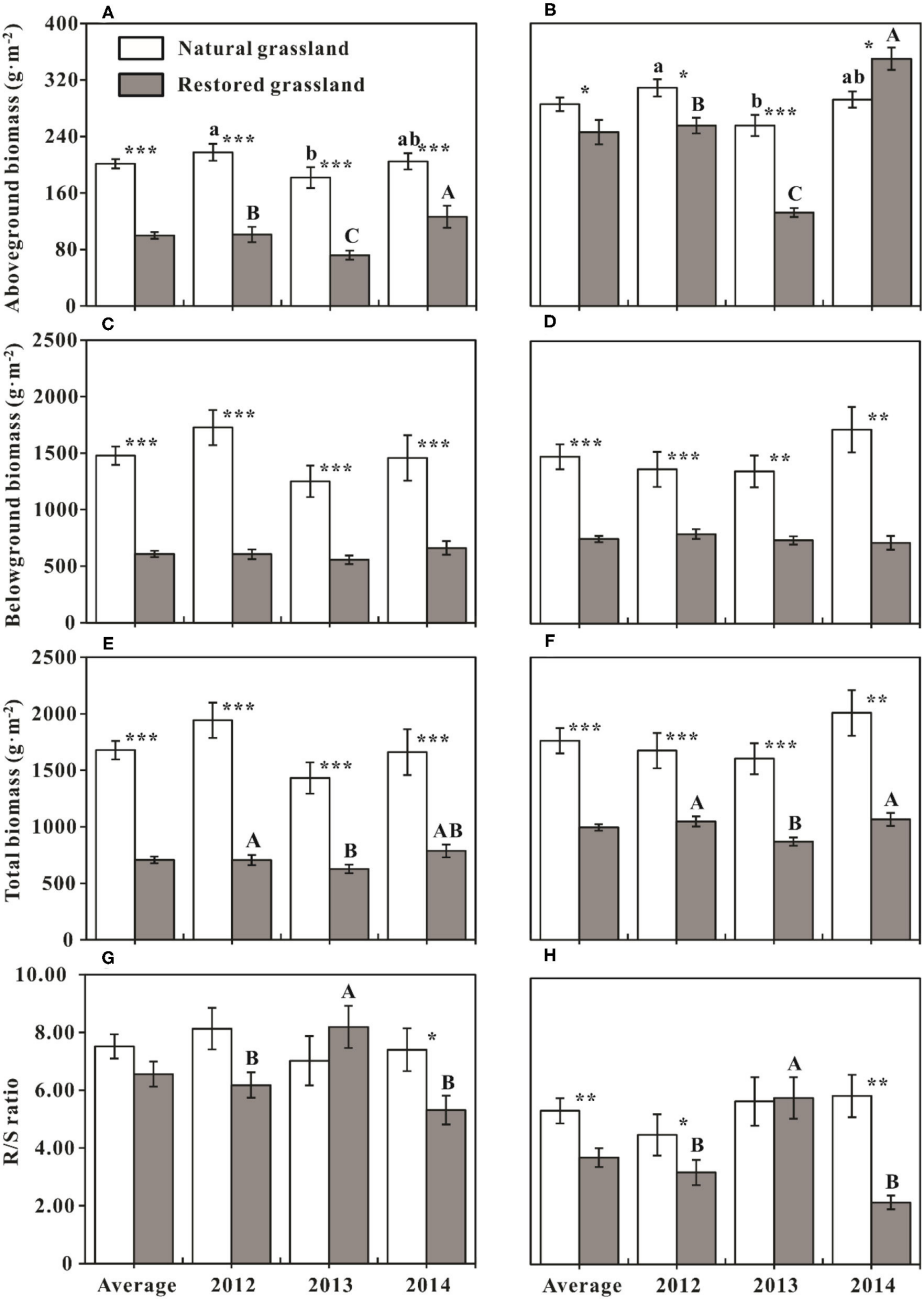
Comparisons of biomass parameters (mean ± SE, *n* = 12) among sampling years in the same grassland and between the natural and restored grasslands in the same years. **(A,C,E,G)** Comparisons of aboveground, belowground, plant community total biomass, and the ratio of root and shoot biomass in treatments without nitrogen addition, respectively. **(B,D,F,H)** Comparisons of aboveground, belowground, plant community total biomass, and the ratio of root and shoot biomass in treatments with nitrogen addition, respectively. Average represents the average effect across the three sampling years (mean ± SE, *n* = 36). Different lowercase letters indicate significant differences of biomass parameters among sampling years in the natural grassland at *p* < 0.05, and uppercase letters indicate significant differences of biomass parameters among sampling years in the restored grassland at *p* < 0.05. *p < 0.05; **p < 0.01; ***p < 0.001, indicate the differences of biomass parameters between the natural and restored grasslands in the same sampling year and same treatment.

Plant AGB of NG was significantly higher than that of RG across different sampling years in treatments without N addition (*p* < 0.001, Figure 2A). In treatments with N addition, AGB of RG was significantly lower than that of NG in the first 2 years of the experiment (*p* < 0.05), whereas significantly higher than that of NG in the 3rd sampling year (*p* < 0.05, Figure 2B). On average, AGB of NG was substantially higher than that of RG in the ambient environment (without N addition, *p* < 0.001, Figure 2A); N addition weakened the difference of AGB between NG and RG (*p* = 0.05, Figure 2B). BGB and TB of NG were significantly higher than that of RG (*p* < 0.01, Figures 2C–F), which was consistent across the 3 experimental years regardless of N addition. Although there was no significant difference in R/S between the two grasslands in the first 2 years of the experiment in treatments without N addition (*p* > 0.05), the R/S of NG was significantly higher than that of RG in 2014 (*p* < 0.05, Figure 2G). In the N addition treatments, R/S of RG was significantly lower than that of NG in 2012 and 2014 (*p* < 0.05, Figure 2H), but no difference was found in 2013 (*p* > 0.05, Figure 2H).

### Interannual Dynamics of Plant Community Biomass and the Influences of Climate Factors

AGB of NG and RG, TB, and the R/S ratio of RG displayed apparent interannual variation (*p* < 0.01, Table 1). Therefore, only the plant biomass parameters with evident year-to-year changes were analyzed.

AGB was lowest in 2013, both in the treatments with and without N addition, and this pattern was consistent between the two grasslands (*p* < 0.05, Figures 2A,B). Compared with NG, the interannual dynamic of AGB in RG was more evident during the whole experiment period, regardless of N addition or not (*p* < 0.05, Figures 2A,B). AGB of RG was highest in 2014, significantly higher than that of the other 2 years; AGB of RG in 2012 was significantly higher than that of 2013 (*p* < 0.05, Figures 2A,B). TB of RG in 2013 was significantly lower than that of the other 2 years (*p* < 0.05, Figures 2E,F), and R/S of RG in 2013 was significantly higher than that of the other 2 years (*p* < 0.05, Figures 2G,H).

**Figure 3:**
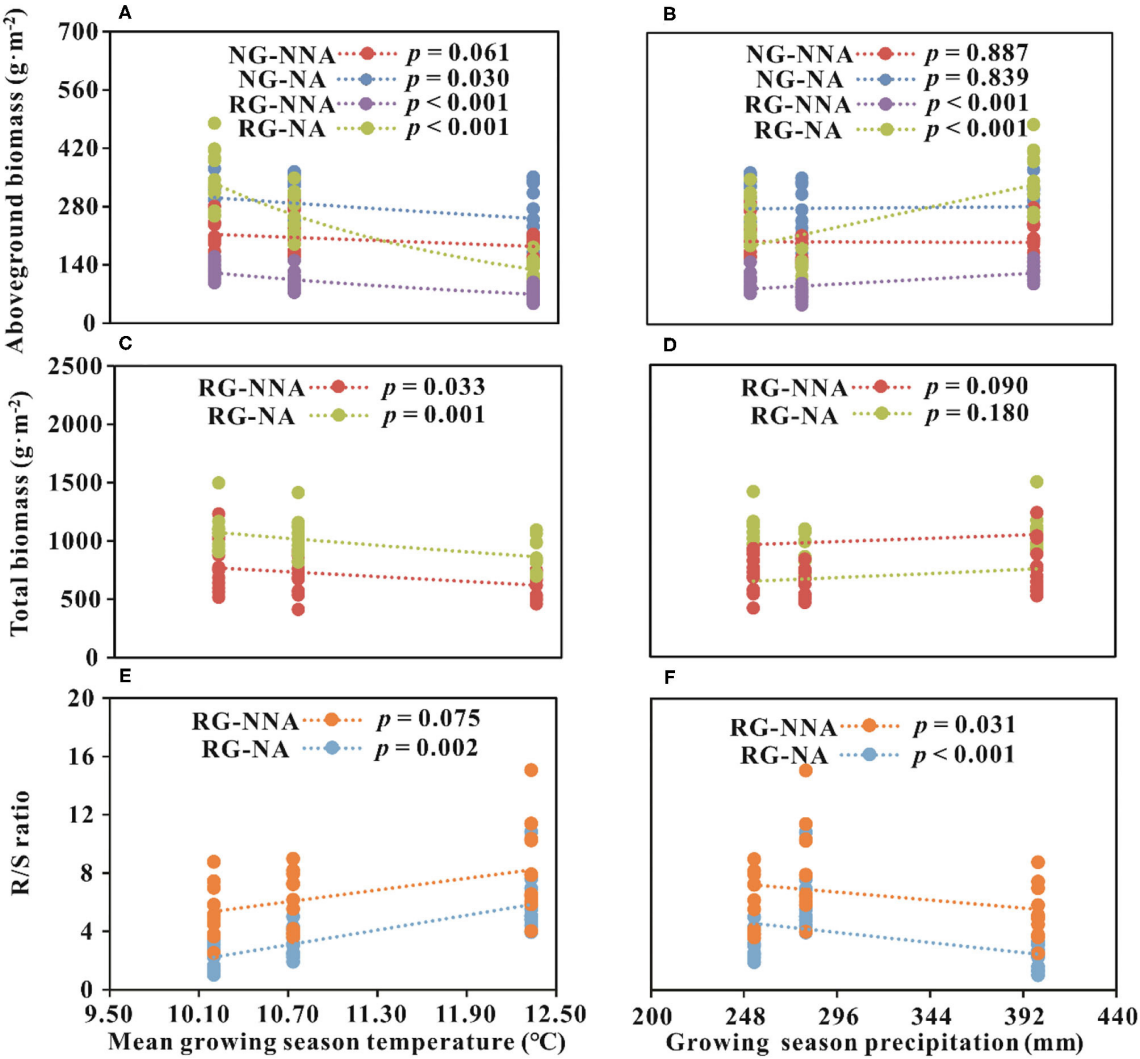
Relationships between biomass parameters and climatic factors. **(A,B)** Relationships between aboveground biomass and climatic factors; **(C,D)** Relationships between total biomass and climatic factors; **(E,F)** Relationships between R/S ratio and climatic factors. R/S ratio, the ratio of root and shoot; NG, natural grassland; RG, restored grassland; NNA, no nitrogen addition; NA, nitrogen addition.

The regression analysis indicated the interannual dynamic of AGB in NG was negatively related to GST with N addition (*p* < 0.05, Figure 3A). Interannual dynamics of AGB in RG was significantly negatively related to GST and was significantly positively associated with GSP, regardless of N addition or not (*p* < 0.001, Figures 3A,B). TB of RG was negatively correlated with GST, but no evident relationship with GSP between the sampling years (Figures 3C,D). The annual fluctuations of the R/S ratio in RG were positively related to GST and negatively associated with GSP (Figures 3E,F).

### Interaction of N Addition With Sampling Year on AGB and R/S in RG

**Figure 4:**
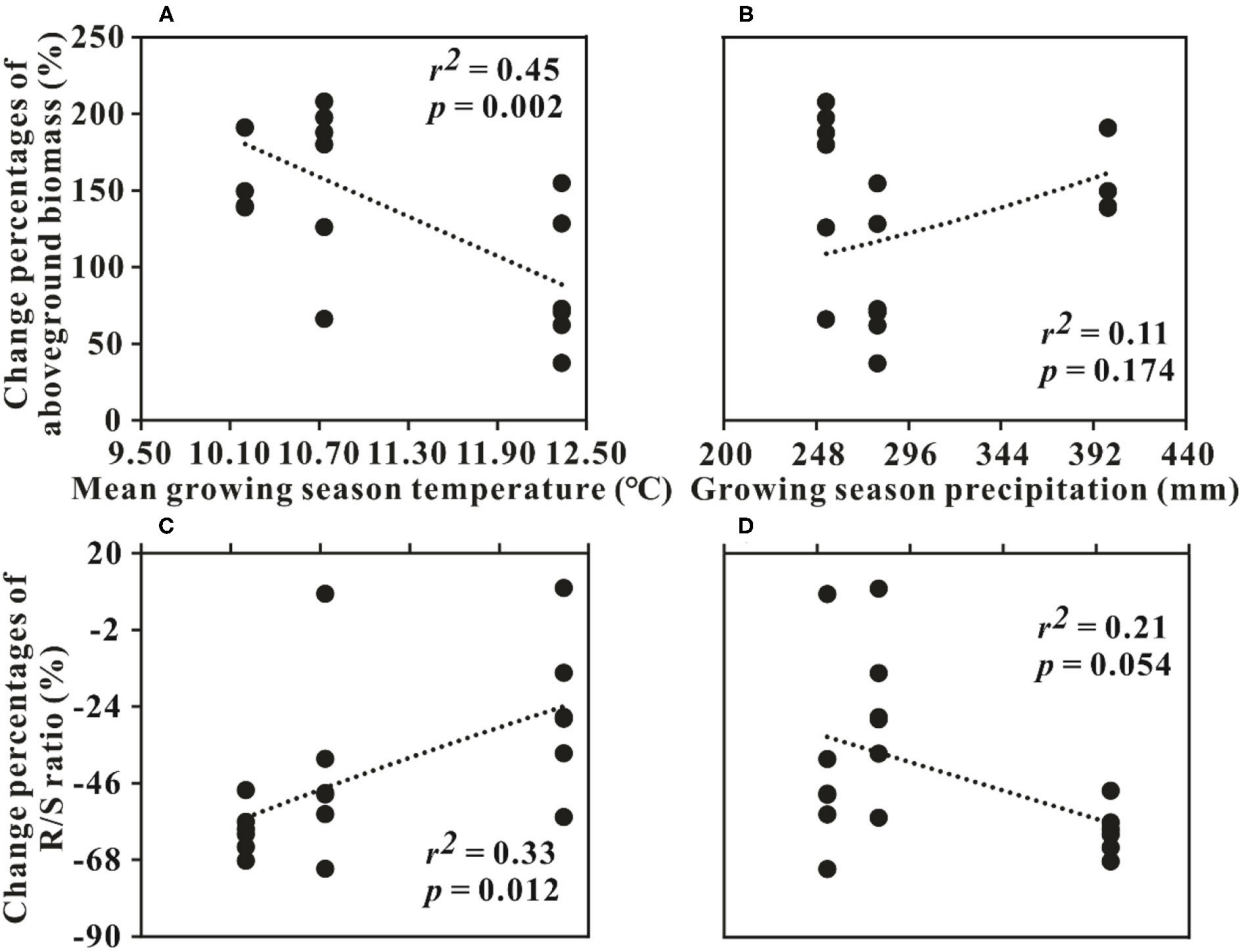
Relationships between the changes in aboveground biomass, **(A,B)** R/S ratio; **(C,D)** and climatic factors in the restored grassland around Qinghai Lake Basin. R/S ratio, the ratio of root and shoot.

The interaction of N addition with the sampling year was found for the AGB and R/S in RG (*p* < 0.05, Table 1). The AGB increment in 2013 was significantly lower than that of the other 2 years (*p* < 0.05, Figure 1A). The changes in AGB were significantly negatively related to GST (*p* < 0.05) but had no relationship with GSP (*p* > 0.05, Figures 4A,B). The decrement of R/S in 2014 was higher than that of the first 2 years (*p* < 0.05, Figure 1D). The reductions of R/S were significantly positively correlated with GST (*p* < 0.05) while showing a weak relation with GSP (*p* = 0.054, Figures 4C,D).

### Correlations Between TB and AGB, TB and BGB, R/S and AGB, and R/S and BGB

No significant correlation was observed between AGB and TB in NG (*p* > 0.05, Table 2). There was a highly positive correlation between BGB and TB (*r* > 0.99, *p* < 0.01, Table 3). For RG, AGB, and BGB both showed positive correlations with TB (*p* < 0.01, Tables 2, 3). However, the Pearson correlations between BGB and TB (*r* = 0.890) were stronger than those between AGB and TB (*r* = 0.630; Tables 2, 3). When all data during the study were pooled, BGB played a vital role in the composition of TB for the alpine grassland (*r* = 0.990, *p* < 0.001), although AGB also played an important role in the form of TB (*r* = 0.450, *p* < 0.001).

**Table 2:** Correlations between plant community total and aboveground biomass in the natural (NG) and restored (RG) alpine grasslands around Qinghai Lake Basin.

**Data analysis**		**Pearson correlation (*r*)**	***p***
**Annual data (*****n*** **=** **24)**			
NG	2012	−0.128	0.551
	2013	0.085	0.692
	2014	0.348	0.096
RG	2012	**0.702**	**<0.001**
	2013	**0.599**	**0.002**
	2014	**0.525**	**0.008**
**Grassland type (*****n*** **=** **72)**			
NG		0.181	0.129
RG		**0.630**	**<0.001**
**All data pool (*****n*** **=** **144)**		**0.450**	**<0.001**

*Significant results (p < 0.05) were bolded*.

**Table 3:** Correlations between plant community total and belowground biomass in the natural (NG) and restored (RG) alpine grasslands around Qinghai Lake Basin.

**Data analysis**		**Pearson correlation (*r*)**	***p***
**Annual data (*****n*** **=** **24)**			
NG	2012	**0.993**	**<0.001**
	2013	**0.992**	**0.007**
	2014	**0.996**	**<0.001**
RG	2012	**0.941**	**<0.001**
	2013	**0.982**	**<0.001**
	2014	**0.847**	**<0.001**
***Grassland type (*****n*** **=** **72)***			
NG		**0.994**	**<0.001**
RG		**0.890**	**<0.001**
**All data pool (*****n*** **=** **144)**		**0.990**	**<0.001**

*Significant results (p < 0.05) were bolded*.

The variations of R/S in NG were correlated with AGB and BGB (*p* < 0.05, Tables 4, 5); but the correlation between BGB and R/S was higher than that of ABG and R/S (Tables 4, 5). The variation of R/S in RG was mainly affected by AGB changes (*p* < 0.001, Table 4), and had no significant relation with BGB (*p* < 0.05, Table 5).

**Table 4:** Correlations between the ratio of root and shoot and aboveground biomass in the natural (NG) and restored (RG) alpine grasslands around Qinghai Lake basin.

**Data analysis**		**Pearson correlation**	***p***
**Annual data (*****n*** **=** **24)**			
NG	2012	–**0.662**	**<0.001**
	2013	–**0.532**	**0.007**
	2014	–**0.421**	**0.041**
RG	2012	–**0.793**	**<0.001**
	2013	–**0.693**	**<0.001**
	2014	–**0.813**	**<0.001**
**Grassland type (*****n*** **=** **72)**			
NG		–**0.518**	**<0.001**
RG		–**0.760**	**<0.001**
**All data pool (*****n*** **=** **144)**		–**0.494**	**<0.001**

*Significant results (p < 0.05) were bolded*.

**Table 5:** Correlations between the ratio of root and belowground biomass in the natural (NG) and restored (RG) alpine grasslands around Qinghai Lake basin.

**Data analysis**		**Pearson correlation (*r*)**	***p***
**Annual data (*****n*** **=** **24)**			
NG	2012	**0.868**	**<0.001**
	2013	**0.846**	**<0.001**
	2014	**0.736**	**<0.001**
RG	2012	0.085	0.694
	2013	0.266	0.209
	2014	**0.446**	**0.029**
**Grassland type (*****n*** **=** **72)**			
NG		**0.781**	**<0.001**
RG		0.167	0.162
**All data pool (*****n*** **=** **144)**		**0.563**	**<0.001**

*Significant results (p < 0.05) were bolded*.

## Discussion

### Effects of Nutrient Additions on Plant Community Biomass in the Alpine Grassland Around QLB

Commonly, nutrient availability limits the primary productivity of grasslands, and fertilization can increase soil quality and nutrient bioavailability, effectively alleviating nutrient restriction and consequently yielding positive effects on plant community productivity of grassland ecosystems (Bracken et al., 2015; Fay et al., 2015; Fu and Shen, 2016a, 2017; Wang et al., 2020). In general, compared with the aggregate responses to N and P addition individually, the responses of grassland primary productivity to N and P addition simultaneously are greater (Harpole et al., 2011; Fay et al., 2015; Cerasoli et al., 2018; Wang et al., 2020). Such previous findings indicate the synergistic co-limitation of N and P on grassland productivity and highlight the critical role of P for primary production. However, the presence and magnitude of nutrient effects depend on site-specific factors, soil development stage, soil fertility, and climate factors (Walker and Syers, 1976; Laliberte et al., 2012).

In this study, N addition promoted AGB considerably, both for NG and RG (Table 1; Figure 1A). The result was consistent with numerous previous studies in grassland ecosystems on a global scale and in the QTB (Borer et al., 2014; Fay et al., 2015; Wang et al., 2015, 2020; Fu and Shen, 2016a; Zhao et al., 2019). BGB in RG increased significantly with N addition (Table 1; Figure 1B). However, P addition did not present significant effects on any plant biomass parameter, and an interaction effect of N and P was also not evident (Table 1). The results implied that N was still the predominant limiting nutrient, and P was less limited for plant growth in the alpine grassland around QLB. This observation was consistent with the conclusion that N limitation generally peaked in cool sites (Fay et al., 2015). Meanwhile, another study also reported that P addition alone did not affect AGB and BGB in a continuous 4-year fertilization experiment in an alpine grassland on the QTP (Luo et al., 2019). However, the results of previous studies and our study were inconsistent with numerous research findings that grassland primary productivity was co-limited by N and P (Grace et al., 2016; Cerasoli et al., 2018; Wang et al., 2020). The following potential reasons might be responsible for the observations.

The properties of N and P determine their degree of limitation on primary productivity. N is regularly bound with the organic substrate by the strongly integrated covalent C–N chemical bond, which requires considerable energy to break apart (McGill and Cole, 1981). Moreover, the primary way for N to enter a terrestrial ecosystem is by fixation of N_2_ by diazotrophs, resulting in a slower biological process for N cycling in natural ecosystems. Soil N mineralization is sensitive to temperature (McGill and Cole, 1981; Booth et al., 2005; van Heerwaarden et al., 2010). By contrast, P is a sedimentary element and enters the terrestrial ecosystem primarily *via* weathering of soil native minerals (Walker and Syers, 1976; van Heerwaarden et al., 2010). Therefore, temperate and cooler ecosystems, such as the QLB with relatively short soil development periods and low temperatures, are more likely to have relatively abundant P and to be limited by N (Yuan and Chen, 2009; Vitousek et al., 2010; Fay et al., 2015). Furthermore, compared with N, P has low mobility and only a tiny part of its surface application to soil could reach the root system (Guo et al., 2016). Generally, soil moisture is positively associated with GSP (Fu et al., 2018). Precipitation around QLB, a semiarid region, is low (~370 mm), which results in lower soil moisture, further lowering the absorption and utilization of P. Thus, additional P could not be sufficiently utilized. This was indicated by the positive correlation between soil P content and moisture in this region (Li et al., 2019). Additionally, P mineralization is closely related to soil pH, and high pH could fix P in the soil and reduce its utilization efficiency (Devau et al., 2009; Li et al., 2019). Therefore, the high pH in this alpine grassland ecosystem might reduce the P availability and the effects of P addition on plant production (Li et al., 2019).

Nevertheless, P addition elevated plant AGB of NG significantly in 2014 (Li et al., 2016b). The following two factors may cause this effect. First, soil available P is significantly positively related to GSP in NG (Li et al., 2019). GSP (399.5 mm) in 2014 was higher than that of the other 2 years (253.2 mm for 2012 and 279.8 mm for 2013). The relatively higher precipitation increased P mobility in soil, allowing plant roots to absorb P. Besides, soil total N (STN) content was likewise positively associated with GSP and significantly increased with N addition in this region (Li et al., 2019). Considering that the soil N background status is the crucial factor affecting soil available N (SAN) in the alpine grassland ecosystem around the QLB, the increasing STN could further mitigate the N limitation in the alpine grassland ecosystems (Fu and Shen, 2017). According to Liebig's Law of the Minimum, plant growth is limited by the most restrictive resource in the ecosystem. Therefore, the responses of vegetation to N and P additions depended on whether limitations from other ecological factors existed after alleviation or elimination of N limitation. STN of NG in 2014 was significantly higher than in the other 2 years (Li et al., 2019). Hence, as N limitation was alleviated with STN elevated significantly, the limiting effect of P was more evident in 2014. Meanwhile, N addition might reduce the soil pH (Fu and Shen, 2017) and accordingly increase the P availability. In terms of the results mentioned above, we predicted that N is the primary limiting element, and the ecosystem responds more rapidly to N addition in the alpine grassland around QLB. Even so, P would become a limiting factor in plant productivity, especially with a warmer and wetter climate change background.

This prediction is consistent with the results of soil of N and P content changes with nutrient addition and temperature and precipitation fluctuations (Li et al., 2019), as well as the finding that plant biomass varied with P addition between wet and dry years (Wang et al., 2018). The conclusion was supported by the result that N addition had a robust promotion effect, but P addition almost had no significant effect on plant community biomass parameters in RG (Table 1). This may be because RG had lower BGB, soil background N values, and moisture, but higher soil P content and pH (Li et al., 2019). However, this prediction needs to be verified over extended periods and in different areas of the QLB.

### Convergent and Divergent Responses of Plant Community Biomass to N Addition and Interannual Dynamics Between NG and RG

In this 3-year nutrient addition experiment, the response of AGB to N addition in RG was higher than that in the NG (Figure 1A). BGB in RG was also improved significantly by 24% with N addition (Table 1; Figure 1B). Meanwhile, plant community total biomass (TB) of RG concurrently increased by 41% on account of the promotion of AGB and BGB with N application (Table 1; Figure 1C). BGB and TB of NG showed no apparent response to N addition (Table 1). This pattern was in line with the results reported by N addition experiments in Inner Mongolia grasslands (Gao et al., 2011). They found the eliminating limiting resource increased the ANPP of intermediately grazed grassland, but the increase was lower than that of heavily grazed grassland (Gao et al., 2011). Additionally, soil N contents (including soil available and total N) in RG were remarkably lower than that of NG at the existing plant community succession stage (Li et al., 2019). Thus, according to the discussion above, we concluded that N was extremely deficient and was the most crucial factor limiting vegetation growth in RG. Therefore, the plant community biomass of RG had a more intense response to N addition. The results indicated that exogenous N input (ongoing and future N deposition) would be conducive to restoring vegetation growth and primary productivity in the degraded alpine grassland around QLB. This prediction could be illustrated by the result that N addition weakened the difference of AGB between NG and RG on average, and AGB of RG was significantly higher than that of NG in 2014 (Figure 2B).

Besides the convergent and divergent responses to nutrient addition, the interannual dynamics of plant community biomass in NG and RG were likewise diverse. Year-to-year variation of AGB was significantly evident in this study, both for the NG and RG (Table 1). Generally, temperature and precipitation are found to regulate plant biomass and affect the response of plant biomass to N addition in alpine grassland ecosystems (Fu and Shen, 2016a; Wang et al., 2020). AGB of 2013 was significantly lower than that of the other two sampling years in the two grassland types (Figures 2A,B). Our results showed that GST had a negative effect, while GSP showed a positive impact on the year-to-year fluctuations of AGB (Figures 3A,B). For NG, the interannual dynamics of AGB were mainly influenced by GST, especially under treatments with N addition (Figure 3A). While, for RG, the interannual dynamics of AGB were significantly impacted both by GST and GSP in the two treatments (Figures 3A,B). As a crucial limiting environmental factor, lower temperature constrains the mineralization of soil N, and it is not advantageous to plant growth and ANPP accumulation in alpine regions (Fu et al., 2015; Fu and Shen, 2016a, 2017; Xu et al., 2016; Chen et al., 2017). Theoretically, warming would promote AGB in the alpine grasslands of the QTP (Fu et al., 2015; Fu and Shen, 2016a; Xu et al., 2016; Chen et al., 2017). However, the intensity and direction of plant response to temperature depend on soil moisture to a certain degree (Chen et al., 2017; De Boeck et al., 2018; Fu et al., 2018, 2019; Li et al., 2018; Xu et al., 2018). Warming could decrease biomass by reducing soil moisture and inhibiting plant growth (Fu et al., 2015, 2019; Zhang et al., 2021), and could increase biomass by promoting organic matter accumulation by enhancing plant uptake of mineral nutrients (Xu et al., 2015a,b; De Boeck et al., 2018; Li et al., 2018). Therefore, there is much uncertainty about the effect of temperature on plant biomass, and the warming effect is partly regulated by soil moisture in the QTP (Fu and Shen, 2016b; Fu et al., 2019; Zhang et al., 2021).

Generally, climate warming would promote plant growth under humid conditions, whereas inhibiting ecosystem C absorption under drought condition (Fu and Shen, 2016b; Li et al., 2018; Quan et al., 2019). In this semiarid ecosystem around QLB, water availability might be another limiting factor restricting plant growth, which resulted in the negative feedback of AGB to GST (Figure 3A). Vegetation coverage in RG (40%) was lower than that of NG (80%; Li et al., 2016b), which would lead to comparatively intense soil evaporation, and climatic warming would subsequently affect soil moisture in RG. Soil moisture of NG changed little between the experimental years and was relatively higher than that of RG (Li et al., 2019). Therefore, the AGB of NG was relatively stable (Figure 2A), and its interannual dynamics were not evidently influenced by GST and GSP in the natural state (Figures 3A,B). In the treatments with exogenous N addition, the rapid growth of plants requires a large amount of water, leading to a significant negative correlation between the annual change in AGB and GST (Figure 3A). A previous meta-analysis in the QTP showed N addition tended to cause warming and drying soil conditions (Fu and Shen, 2016a). This might be the other reason that GST had a negative effect on the AGB. For RG, lower plant coverage resulted in soil moisture of RG in 2013 was significantly lower than in the other 2 years (Li et al., 2019). Consequently, the fluctuation of AGB was notable (Figures 2A,B), and impacted greatly by GST and GSP (Figures 3A,B). The results implied the importance of precipitation to the restoration of degraded alpine grasslands and was supported by the other studies on the QTP (Sun et al., 2019; Wei et al., 2019). In sum, our study could provide profound insight into exploring the responses of alpine grasslands to the ongoing and future climate change with land use on the QTP.

Compared with AGB, BGB was comparatively stable, with minor changes year to year (Table 1; Figures 2C,D). Plant community TB was predominantly driven by BGB (Table 3) and was unaffected by AGB in NG (Table 2). This further resulted in stable intra-annual changes of TB in NG among sampling years (Table 1). For RG, AGB and BGB both significantly contributed to TB (Tables 2, 3). Therefore, TB of RG showed significant intra-annual variation among sampling years (Table 1) and was significantly affected by GST (Figure 3C). A weak relationship between TB and GSP in RG likely resulted from BGB accounting for more TB. Besides, interannual variations in meteorological factors likely changed the effects of N on AGB among sampling years. In RG, AGB was negatively related to GST while positively related to precipitation, suggesting the effects of climate change on plant primary production are a trade-off between warming and precipitation change. This trade-off is likely to be regulated by soil moisture (Fu and Shen, 2016a; Fu et al., 2018; Wang et al., 2020). Based on the preceding results, we concluded that climate warming would reduce soil moisture, and our study showed a sequentially indirect negative effect on plant community AGB and TB. Moisturizing (including precipitation and soil water content increasing) would be beneficial to enhance ANPP during the restoration of grasslands.

### Plant Community Biomass Allocation Patterns in NG and RG

As the major component of plant biomass and the C pool, BGB is central to the functioning of terrestrial ecosystems, related to ecosystem nutrient, water, and C cycling (Bardgett et al., 2014; Ottaviani et al., 2020). On a global scale, belowground biomass accounts for ~67% of grassland ecosystems (Ma et al., 2021). In our study, the proportion of root biomass approached 85% in the surface soil layer in the natural state (without N addition), resulting in a relatively high R/S (around 7), both for the NG and RG. This value was higher than the global level but in line with the conclusion that more biomass will be allocated to the underground roots. This represents a trade-off in resource allocation in cold and dry ecosystems because nutrient supply and water availability are lower in these regions (Reich et al., 2014; Ledo et al., 2018; Qi et al., 2019; Ma et al., 2021).

With nutrient enrichment, the R/S ratio usually decreased (Peng and Yang, 2016). The R/S variation reflected the functional balance between the distribution of resources that exist above and below (water, nutrients) the soil surface (Chen and Reynolds, 1997; Franklin et al., 2012). In this study, N addition reduced the R/S ratio significantly by 28 and 35% for the NG and RG, respectively (Figure 1D). This was consistent with previous studies (Peng and Yang, 2016). R/S ratio variations of NG were associated with AGB and BGB, and more correlated with BGB (Tables 4, 5). This result might explain the stability of R/S among sampling years (Table 1).

The R/S ratio changes of RG were only dependent on AGB (Table 4), and had little correlation with BGB (Table 5). This pattern could generate significant inter-annual dynamics of the R/S ratio in RG (Table 1). Additionally, interannual variations in meteorological factors likely changed the effects of N on the differences of the R/S ratio among years. The R/S ratio and the decrement of R/S ratio were positively associated with GST and negatively with precipitation, suggesting that climate warming may exacerbate water limitation and would cause more plant biomass to be allocated to underground roots for absorbing more nutrients and water. Increasing precipitation could be conducive to the growth of plant aboveground part. It was totally in line with the previous outcomes (Ma et al., 2021). Combined with the preceding analyses, our results indicated that climate change (warming and precipitation increasing) could affect plant community biomass accumulation and allocation mainly by regulating soil moisture in the alpine grasslands around QLB.

However, interestingly, the R/S in the two grasslands did not show a significant difference in the natural state (Figure 2G), although soil nutrient and moisture were most pronounced in NG (Li et al., 2019). That is, the allocation of plant biomass between above- and belowground was similar in the NG and RG. The results indicated that the RG was also in a relatively stable state after a 10-year restoration. Given the above results, higher AGB might signify a higher BGB for maintaining a stable R/S ratio and balancing above- and belowground biomass.

## Conclusions

This study determined the various responses of plant community biomass to N and P additions in NG and RG by a 3-year *in situ* manipulation experiment around the QLB. Our results suggested that the ongoing N deposition would benefit plant growth and primary productivity for the alpine grassland around QLB under future climate warming and precipitation scenarios, especially by implementing the “Grain for Green” project.

## Data Availability Statement

The raw data supporting the conclusions of this article will be made available by the authors, without undue reservation.

## Author Contributions

CL collected and analyzed the data and wrote the original draft. CZ analyzed the data, reviewed, and reorganized the manuscript. YL, XL, LM, and YX participated in discussions. All authors contributed to the article and approved the submitted version.

## Funding

This study was supported by the Fundamental Research Project of Qinghai Province (2019-ZJ-935Q), the Open Project of State Key Laboratory of Plateau Ecology and Agriculture, Qinghai University (2018-ZZ-02, 2020-ZZ-07), the Joint Research Project of Three-River- Resource National Park funded by Chinese Academy of Sciences and Qinghai Provincial People's Government (LHZX-2020-08). CZ was supported by the 1,000 Talent program of Qinghai Province.

## Conflict of Interest

The authors declare that the research was conducted in the absence of any commercial or financial relationships that could be construed as a potential conflict of interest.

## Publisher's Note

All claims expressed in this article are solely those of the authors and do not necessarily represent those of their affiliated organizations, or those of the publisher, the editors and the reviewers. Any product that may be evaluated in this article, or claim that may be made by its manufacturer, is not guaranteed or endorsed by the publisher.
